# Toxicity and Dermatokinetic Analysis of Ibrutinib in Human Skin Models

**DOI:** 10.3390/pharmaceutics16111377

**Published:** 2024-10-26

**Authors:** Maria Victória Souto-Silva, Elizabete C. I. Bispo, Lucas F. F. Albuquerque, Stefhani Barcelos, Emãnuella M. Garcez, Luana S. Quilici, Florêncio Figueiredo Cavalcanti Neto, Eliza Carla Barroso Duarte, Jankerle N. Boeloni, Felipe Saldanha-Araujo, Guilherme M. Gelfuso, Juliana Lott Carvalho

**Affiliations:** 1 Interdisciplinary Biosciences Laboratory, Faculty of Medicine, University of Brasilia, Brasília 70910-900, DF, Brazil; 2 Laboratory of Hematology and Stem Cells, Faculty of Health Sciences, University of Brasilia, Brasília 70910-900, DF, Brazilfelipearaujo@unb.br (F.S.-A.); 3 Laboratory of Food, Drugs and Cosmetics (LTMAC), Faculty of Health Sciences, University of Brasilia, Brasília 70910-900, DF, Brazilgmgelfuso@unb.br (G.M.G.); 4 Catholic University of Brasilia, Brasília, DF, 71966-900, Brazil; 5 Department of Pathology, Faculty of Medicine, University of Brasilia, Brasilia 70910-900, DF, Brazil; 6 Center for Agricultural Sciences and Engineering, Department of Veterinary Medicine, Federal University of Espírito Santo, Alegre 29500-000, ES, Brazil

**Keywords:** hOSEC model, alternative models, melanoma, skin permeation, SKMEL-28

## Abstract

**Background/Objectives:** Ibrutinib (IBR) is a tyrosine kinase inhibitor under investigation in preclinical and clinical settings as an alternative treatment for melanoma. Nevertheless, the limited oral bioavailability of IBR and the need for high doses of the drug to kill melanoma cells are major drawbacks for this purpose. Considering that melanoma is restricted to the skin at early stages, the topical application of IBR might constitute an effective and safer administration route. In this study, we determined IBR’s toxicity and dermatokinetics using human primary cells and human organotypic skin explant cultures (hOSECs). **Methods:** After demonstrating that human primary fibroblasts and keratinocytes present IBR target genes, the cytotoxicity of the drug was determined using the MTT and annexin V/PI staining assays. IBR toxicity in the skin was assessed using the TTC assay, and the irritation potential was established using histological assessment. Finally, IBR cutaneous permeation was assessed ex vivo to determine the drug dermatokinetics. **Results:** Our findings reveal that IBR exerts dose-dependent toxicity towards skin cells, presenting an IC_50_ in the same range as melanoma cells. The topical application of the drug successfully reduced irritation and toxicity in the skin, and the drug was shown to successfully permeate the stratum corneum and reach the viable skin layers in therapeutic concentrations. **Conclusions:** Overall, our data encourage the topical application of IBR to treat melanoma, paving the way for future studies in this theme.

## 1. Introduction

Normal melanocytes protect the skin against ultraviolet radiation and regulate pigmentation. However, when there is dysfunction in these melanocytes, abnormal proliferation and transformation into malignant melanoma can occur [1]. From a molecular perspective, melanoma is characterized by various somatic mutations that can drive disease progression. These changes generally affect genes like *BRAF* (B-raf proto-oncogene) and *NRAS* (Neuroblastoma RAS Viral Oncogene Homolog), which are essential in cell proliferation, growth, metabolism, cell identity determination, the control of apoptosis, the regulation of the cell cycle, and senescence. The accumulation of such mutations follows specific patterns, giving rise to different pathways of disease progression and resulting in distinct subtypes of melanoma [2].

Melanoma represents the most aggressive and challenging variant of skin cancer [3], frequently evolving into a highly invasive and metastatic condition [4]. Despite comprising only 1% of skin cancer cases, it is responsible for more than 80% of fatalities related to this cancer type [5,6].

Treatment success is heavily influenced by the stage in which melanoma is diagnosed, with localized lesions presenting a more favorable 5-year relative survival rate [7]. Local resection remains the primary method of treatment for melanoma. Nevertheless, combined treatments are indicated to improve clinical outcomes, with chemotherapy marking an important advance in melanoma treatment despite adverse reactions and resistance [8]. Consistent therapeutic advances have been observed in other types of solid tumors and onco-hematological diseases, primarily using the tyrosine kinase inhibitor ibrutinib (IBR) [9]. IBR (1-[(3R)-3-[4-amino-3-(4-phenoxyphenyl)-1H-pyrazolo [3,4-d]pyrimidin-1-yl]- 1-piperidinyl]-2-propen-1-one) is one of the few tyrosine kinase inhibitors that has been explored in the treatment of solid and non-solid cancers, with Bruton’s tyrosine kinase (BTK) being its main target [10,11]. IBR acts mainly (but not only) on BTK by binding to the amino acid Cys 481 in the ATP-binding domain. BTK is essential for the antigen receptor signaling pathway in B cells. Furthermore, IBR inhibits IL-2-inducing kinase (ITK), compromising the activation of the nuclear factor kappa B (NF-KB) pathway. It is important to note that the target cysteine residue of IBR is also present in other protein tyrosine kinases, allowing for its action on several other molecular targets, such as members of the Tec family kinases, epidermal growth factor receptor (EGFR) family kinases, and other kinases, such as B lymphoid kinase (BLK) and Janus Kinase (JAK3) [12].

IBR is approved by the Food and Drug Administration (FDA) to treat mantle cell lymphoma, chronic lymphocytic leukemia, Waldenstrom macroglobulinemia, marginal zone lymphoma, and chronic graft-versus-host disease after stem cell transplantation, among others [11]. Generally well tolerated, IBR offers quick and long-lasting responses, although it can cause side effects, such as diarrhea, upper respiratory tract infection, bleeding, fatigue, and cardiac side effects [11]. Dermatological events related to IBR are frequent but are generally mild to moderate in intensity. Skin toxicities associated with IBR include skin irritation, hematomas, petechiae, ecchymosis, skin abscesses, peripheral edema, stomatitis/mucositis, and, in severe cases, hemorrhagic events [13,14]. Although the therapeutic effect has been shown to be effective, there is a therapy discontinuation rate of between 11% and 50% [11].

The adverse reactions to IBR can be explained, in part, by the restricted oral bioavailability due to its intense first-pass metabolism and low solubility, leading to the use of high-dose pharmaceutical forms [15]. In this context, the topical application of IBR might constitute an effective and safe administration route of this drug for early-stage melanoma treatment, as already suggested in the literature for certain chemotherapy drugs [16]. The non-systemic application of the drug could possibly bring the benefit of reducing or completely eliminating side effects related to oral administration without compromising the penetration of the drug in the site of interest. Nevertheless, at this point, there is no literature detailing the toxicity and irritation profile of IBR to the human skin. Furthermore, IBR skin absorption has only been recently described by our group in an animal model in vitro [17].

In this study, we aimed to assess the safety and dermatokinetic profile of topically administered IBR. First, we demonstrated that the main skin cell types—fibroblasts and keratinocytes—express genes that are targeted by IBR and that the drug has a dose-dependent toxic effect on these cells. Next, we used human organotypic skin explant cultures (hOSECs) to evaluate the drug’s toxicity in more realistic models of human skin. Our findings showed that the topical application of IBR effectively reduced skin toxicity and that the drug penetrates the stratum corneum for at least 24 h, reaching viable skin layers at therapeutically relevant concentrations. Thus, this study may support the use of IBR as a topical treatment for melanoma in situ.

## 2. Material and Methods

### 2.1. Material

All reagents were obtained from Sigma-Aldrich (St. Louis, MO, USA), Thermo Fisher Scientific (Waltham, MA, USA), Cayman Chemical (Ann Arbor, MI, USA), and BD Biosciences (East Rutherford, NJ, USA), unless stated otherwise.

### 2.2. Experimental Design

**Figure 1:**
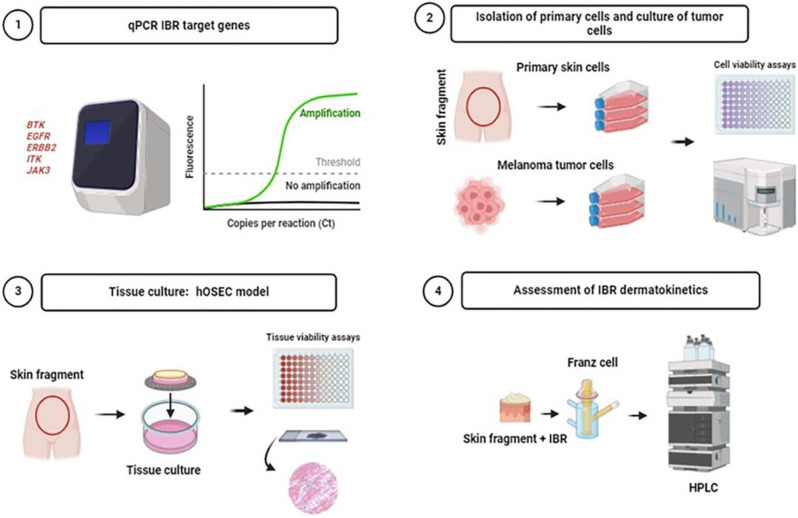
Experimental design. This study included 4 different phases. 1. qRT-PCR of IBR target genes was performed to evaluate the expression of these genes in primary human skin and melanoma cells. 2. Human primary keratinocytes and fibroblasts were isolated and treated with increasing concentrations of IBR to carry out MTT metabolization and apoptosis detection assays. Melanoma cells were also investigated. 3. hOSEC models were used to determine the toxicity and irritation profile of IBR applied topically and in the tissue culture media at different concentrations. The TTC metabolization assay was performed, and the skin irritation score was determined using histology. 4. Assessment of IBR dermatokinetics in hOSECs. Drug permeation was determined after 12 and 24 h of IBR application using HPLC.

In this study, the toxic effects of IBR on human primary fibroblasts and keratinocytes were investigated through cell viability and apoptosis tests. Primary cells obtained via isolation from human skin were used, with experiments carried out using at least three biological and technical replicates. As a comparative parameter, the toxicity of the drug was also determined in cells of the SKMEL-28 tumor lineage, acquired from American Type Culture Collection (ATCC). After this step, the toxicity and irritation of IBR in hOSECs were determined using tissue viability and histological irritation tests. Finally, we analyzed the skin permeation of IBR using the hOSEC model to determine its dermatokinetics (Figure 1). The methods performed in each experiment are described below.

### 2.3. IBR Dilution

The IBR (Cayman Chemical, Cat. #16274, Ann Arbor, MI, USA) solution used in all experiments was prepared by first dissolving the drug using DMSO (Sigma-Aldrich, Cat. #D4540, St. Louis, MO, USA) at 2000 µM, and then by diluting the drug using cell culture medium. For the skin permeation assay, IBR was prepared using Carbopol^®^ 940. Initially, 0.5 g of Carbopol was dispersed in 90 mL of Milli-Q water and allowed to rest for 12 h. Afterwards, triethanolamine was added, and the volume was adjusted to 100 mL using water. Subsequently, 1 mg/mL of IBR was incorporated.

### 2.4. Isolation and Culture of Primary Skin Cells

Keratinocytes and fibroblasts were isolated from fragments of human skin that was taken from healthy patients undergoing abdominoplasty procedures. The procedure was approved by the research ethics committee of the University of Brasília (Protocol n. 30175020.0.0000.5558). Samples were obtained after consent was obtained from patients.

Following hypodermis removal, the tissue was fragmented into small pieces and treated with a dispase solution that was prepared by diluting the enzyme in phosphate-buffered saline (PBS) at 4 mg/mL (Gibco^TM^, Cat. #17105041, Waltham, MA, USA). After overnight incubation at 4 °C, the dispase solution was inactivated, and the dermis was separated from the epidermis with the aid of sterile scissors and tweezers. Digestion of the epidermis was performed with 0.05% trypsin (prepared by diluting a stock solution of 2.5% trypsin, Gibco^TM^, Cat. #15090–046, in PBS) for 10 min to isolate the keratinocytes. The digestion of the dermis was performed with type II collagenase (Gibco^TM^, Cat. #17100017), which was prepared by diluting the enzyme in PBS at 1 mg/mL for 3 h to obtain fibroblasts.

The fragments resulting from the epidermis digestion were filtered through a 100 µM cell strainer (Corning^®^, Cat. #CLS431752, Corning, NY, USA) and centrifuged at 1200 rpm for 4 min. The cells were resuspended in Keratinocyte Serum-Free Medium (KSFM) (Gibco^TM^, Cat. #17005042) and plated at 1 × 105 cells/cm^2^. For fibroblasts, after digestion, collagenase was inactivated using Dulbecco’s Modified Eagle Medium (DMEM) (Gibco^TM^, Cat. #12100046) supplemented with 10% fetal bovine serum (FBS; Gibco^TM^, Cat. #12657029). The fragments were then centrifuged at 2000 rpm for 10 min, the supernatant was discarded, and cells were cultured using DMEM supplemented with 10% FBS and antibiotics (100 U/mL; Gibco^TM^, Cat. #15140122). The cells were expanded upon reaching approximately 90% confluency. The keratinocytes from passages 1 to 3 and fibroblasts from passages 3 to 5 were used in the experiments.

### 2.5. qRT-PCR Detection of IBR Target Genes

The total RNA was isolated from keratinocytes, fibroblasts, and SKMEL-28 cells using Trizol reagent (Invitrogen^TM^ Cat. #15596026, Waltham, MA, USA), following the manufacturer’s instructions. The RNA concentration was determined via an absorbance reading at 260/280 nm on the Nanodrop One (Thermo Scientific, San Jose, CA, USA). The integrity of the isolated RNA was confirmed via electrophoresis on a 1% agarose gel. The samples were then reverse transcribed using the High-Capacity Reverse Transcription kit (Applied Biosystems^TM^; Cat. #4368814, Waltham, MA, USA) according to the manufacturer’s instructions.

The transcript levels of *EGFR*, *JAK3*, *BTK*, *ITK*, and *ERBB2* mRNA were determined using specific primers (Table 1) and KiCqStart^®^ SYBR^®^ Green qPCR ReadyMix (Sigma-Aldrich; Cat. #KCQS01). The calibrator gene used was glyceraldehyde-3-phosphate dehydrogenase (*GAPDH*), allowing for comparative analysis using the 2(-DDCt) method.

### 2.6. Cell Viability Assays

#### 2.6.1. MTT Assay

The viability of keratinocytes, fibroblasts, and melanoma cells from the SKMEL-28 line was assessed using the colorimetric 3-[4,5-dimethylthiazol-2-yl]-2,5-diphenyltetrazolium bromide (MTT) metabolization method, which is based on the ability of viable cells to reduce tetrazolium salt in formazan crystals [18]. A total of 7 × 10^4^ cells were seeded in 96-well culture plates. After 24 h of plating, cell adherence was checked, and the medium was replaced with fresh culture medium (KSFM, for keratinocytes; DMEM supplemented with FBS and antibiotics for fibroblasts and SKMEL-28 cells) containing IBR at different concentrations (0, 1, 2, 5, 10, 25, 50, and 100 µM). After 48 h of incubation, the culture medium was exchanged again for 90 µL of fresh medium plus 10 µL of MTT solution (Invitrogen^TM^, Cat. #M6494), which was prepared by diluting the reagent in PBS, at 5 mg/mL. After incubating the plates for 4 h at 37 °C, 5% CO_2_, and protected from light, 100 µL of dimethyl sulfoxide (DMSO) was added to dissolve the formazan crystals. The absorbance was measured at 540 nm [19]. The IC_25_ and IC_50_ values were calculated for each cell type using nonlinear regression analysis in GraphPad Prism 9 (GraphPad Software, Inc., San Diego, CA, USA).

#### 2.6.2. Apoptosis Detection Assay

The assay was conducted using flow cytometry, and two dyes were used to evaluate cell death: annexin V (BD Pharmingen™ FITC Annexin V; Cat. # 556420, Franklin Lakes, NJ, USA) and propidium iodide (PI; Invitrogen^TM^, Cat. #P3566, Waltham, MA, USA). For this, 8 × 10^5^ cells were plated, and after 24 h of incubation for adhesion to the culture surface, the culture medium was changed to fresh culture medium with IBR added at IC_25_ and IC_50_ concentrations. After 48 h of incubation, the cells were harvested, collected, and stained with the respective dyes, according to the manufacturer’s instructions. Ten thousand events from each sample were recorded using the Attune^®^ NxT Acoustic Focusing Cytometer flow cytometer (Thermo Fisher Scientific, Waltham, MA, USA). Cells that were negative for annexin V and PI were considered viable, while those positive for annexin V and negative for PI, as well as those positive for both dyes, were considered apoptotic. The data were analyzed using FlowJo 10.0.7 software (Treestar, Inc., Ashland, OR, USA) [20].

### 2.7. Human Organotypic Skin Explant Culture (hOSEC)

Healthy skin explants were obtained from healthy abdominoplasty patients who signed the consent form prior to the procedure. The study protocol was approved by the research ethics committee of the University of Brasília prior to initiation (Protocol n. 30175020.0.0000.5558).

**Figure 2:**
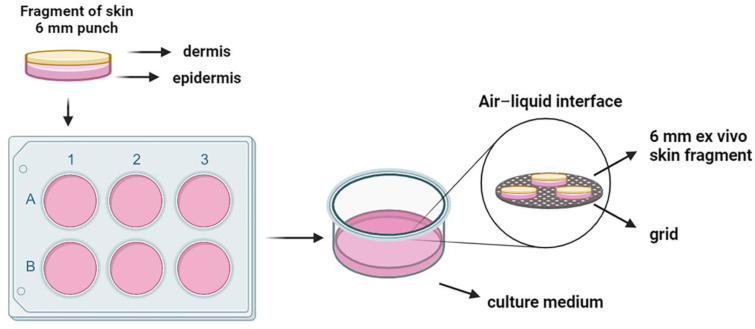
Human organotypic skin explant cultures. Skin fragments obtained from healthy donors were processed and cut into circular 6 mm fragments. The explants were maintained in an air–liquid interface for up to 7 days.

The skin was disinfected, and the hypodermis was removed. Then, it was sectioned with 6 mm punches. The fragments were positioned on top of a sterile metal grid in 6-well plates, maintaining the dermis in contact with the culture medium (DMEM supplemented with 10% FBS and 1% penicillin/streptomycin) and the epidermis in contact with the air (Figure 2). The fragments were incubated at 37 °C and 5% CO_2_ [19].

### 2.8. Tissue Viability Assay

#### 2.8.1. TTC Metabolization Assay

To evaluate the viability of hOSEC, a colorimetric method of 2,3,5-triphenyl-2H-tetrazolium chloride (TTC; Sigma-Aldrich, Cat. #T8877) metabolization was used. The assay serves as an indicator of the metabolic state of the tissue and is used to distinguish between metabolically active and inactive parts [21]. To do so, skin preparation was carried out as previously described. The skin fragments were then topically treated with 25 µL of IBR at different concentrations and treated with the addition of IBR to the medium (0, 10, 25, 50, and 100 µM). As a negative viability control, two experimental groups were added, consisting of skin fragments treated with 50% DMSO and 20% sodium dodecyl sulfate (SDS) (Sigma-Aldrich, Cat. #L3771). The treatments were maintained for 48 h. After the incubation period, the fragments were transferred to a 24-well plate, with one fragment per well. Then, 1 mL of a 2% TTC solution diluted in DMEM without phenol red (Gibco^TM^, Cat. #31053028) was added to each well, and the plate was incubated again for 2 h. After this step, the fragments were washed with PBS and then with a mixture of 70% ethanol and DMSO solution (1:1, *v*/*v*). The extraction was carried out overnight, at room temperature, on a plate shaker protected from light. Subsequently, 200 µL samples were taken from each well and transferred to a 96-well plate, where the absorbance was measured at 485 nm.

#### 2.8.2. Histological Assessment of Skin Irritation

Histological alterations of hOSECs were observed after the skin fragments were topically treated with different concentrations of IBR (0, 10, 25, 50, and 100 µM). As positive irritation controls, the samples were treated with DMSO 50% or SDS 20% and were diluted using sterile water. After 48 h, the skin fragments were fixed in 10% formalin and embedded in paraffin. They were dehydrated in ethanol baths of increasing grade and cleared with xylene before being included in the paraffin and were stained with Hematoxylin and Eosin, as well as Masson’s Trichrome, using standard protocols. Then, tissue sections were analyzed by a pathologist who was blind to the experimental groups. The results of the histological analysis were determined as described by Matarrese et al. [22], under the supervision of a pathology specialist. All evaluations were carried out independently by a pathologist who was blinded to the experimental groups, guaranteeing impartiality in the analyses. The transverse sections were thoroughly examined, showing a variety of possible changes, and were classified on a scale of 0 to 4. Specific criteria were established for the controls to validate the results of the experiment as follows: (1) The histological quality of the untreated controls should be good enough to allow for detailed analysis. (2) Untreated controls should present similar histological results, with possible changes such as exocytosis with or without spongiosis or peri-nuclear edema mainly attributable to preparation artifacts. However, such changes should not exceed grade 2 and/or compromise more serious interpretations. (3) Death-positive controls (treated with DMSO or SDS) should exhibit more severe changes than untreated, negative controls. (4) The severity of these changes should be dose dependent, and at the highest dose (100 µM), the skin explants should present pre-necrotic or intense necrotic lesions.

These criteria were established to ensure the reliability and relevance of the results obtained in the histological analysis of skin irritation, thus providing a solid basis for the interpretation of the data [22] (Table 2).

### 2.9. IBR Skin Permeation and Dermatokinetic Analyses

The skin preparation procedure was conducted as explained previously. Unlike previous experiments, the fragments were cut into circles with an area of approximately 2 cm² and stored at 4 °C to be used later within 30 days. The concentration of IBR used in the experiment was 1 mg/mL (2.27 mM). For this experiment, the drug was suspended in a Carbopol gel, as described. The fragments were placed in Franz vertical diffusion cells, which were positioned between the donor and receptor compartments. The receptor compartment was filled with 15 mL of PBS and supplemented with 30% ethanol to ensure sink conditions (IBR solubility in the receptor media = 174 µg/L) [17], while the donor compartment was filled with 500 µL of IBR at 1 mg/mL. The system was maintained at 37 °C using a thermal bath, with moderate agitation (300 rpm) for 12 and 24 h. At the completion of the experiment, the stratum corneum (SC) and the remaining skin (RS) were separated using the tape-stripping technique. Briefly, the skin fragments were cleaned with water to remove the IBR solution and fixed on a foam support with the SC facing upwards. We used 15 adhesive tapes to remove the SC, fixing each tape over the tissue and removing it with a single movement. Then, the RS fragments were cut into small portions. Each layer was placed individually in amber bottles containing 5 mL of methanol for drug extraction, maintaining moderate agitation (300 rpm) for 24 h. The extracts were subsequently taken for High-Performance Liquid Chromatography (HPLC) analysis using a C_18_ reversed-phase column (150 mm × 4.5 mm, particle size = 5 µm), with a mobile phase composed of acetonitrile (Sigma-Aldrich, Cat. #439134) and acidified water (0.01 M phosphoric acid; Supelco, Cat. #49685) in a ratio of 35:65 (*v*/*v*). The flow rate was 1.0 mL/min, the sample injection volume was 20 µL, the column oven was maintained at 35 °C, and the drug was detected at 359 nm, with an average IBR retention time of 8.9 min. Shimadzu LC software, version 5.99, was used to acquire, analyze, and generate reports on the data obtained, having been previously validated by our group [23].

### 2.10. Data Analysis

Histological analysis data were analyzed qualitatively. The other results were analyzed quantitatively. The data are presented as the mean ± standard deviation (SD) or standard error. The differences between the amounts released or penetrated of the drug from each formulation were analyzed using the ANOVA. The Tukey test was then performed when statistically significant differences between means were detected in the ANOVA test. The variables “toxicity”, “migration”, and “gene X expression” were tested for normality using the Lilliefors test. Variables that had a normal distribution were analyzed using the ANOVA and Tukey or Bonferroni post hoc tests. Statistical significance was set at *p* < 0.05.

## 3. Results

### 3.1. Human Primary Skin Cells Present IBR Target Genes

**Figure 3:**
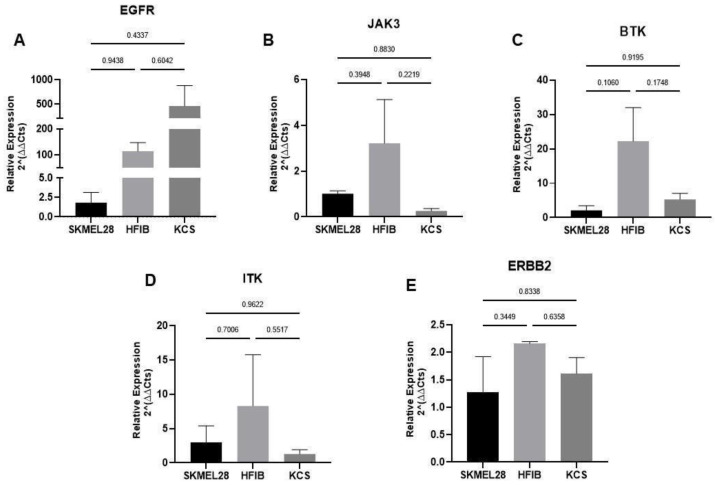
qRT-PCR of IBR target genes in primary cells from the skin. The mRNA levels of EGFR (**A**), JAK3 (**B**), BTK (**C**), ITK (**D**), and ERBB2 (**E**) were determined in human primary fibroblasts (HFIB) and keratinocytes (KCS). The median Ct values obtained from SKMEL-28 cells were used as a reference. All real-time PCR reactions were performed in technical duplicate. The relative fold change was obtained using the formula 2−ΔΔCt, using GAPDH to normalize sample loading. No comparisons between the groups resulted in statistically significant differences. The data were normalized to SKMEL-28 mRNA levels and are represented with the mean ± standard error of the mean (SEM). The data were analyzed using the ANOVA and Tukey post hoc test.

We first determined whether human primary keratinocytes and fibroblasts present IBR target genes, namely *EGFR* (Figure 3A), *JAK3* (Figure 3B), *BTK* (Figure 3C), *ITK* (Figure 3D), and *ERBB2* (Figure 3E). Through qRT-PCR, we have shown that both primary cell types present variable transcript levels of the main IBR target genes, with no statistically significant differences being detected among the cell lines. Compared to SKMEL-28, hFibs displayed a mean mRNA expression difference of 114-fold higher in *EGFR*, 3.2-fold higher in *JAK3*, 22.2-fold higher in *BTK*, 8.3-fold higher in *ITK*, and 2.1-fold higher in *ERBB2*. In contrast, keratinocytes exhibited a 459-fold higher mean mRNA expression in *EGFR* while showing a 0.7-fold decrease in *JAK3*, a 3.2-fold higher in *BTK*, a 1.7-fold decrease in ITK, and a 0.3-fold higher in *ERBB2* relative to SKMEL-28.

### 3.2. Human Primary Skin and Melanoma Cells Present Different Levels of Sensitivity Towards IBR

**Figure 4:**
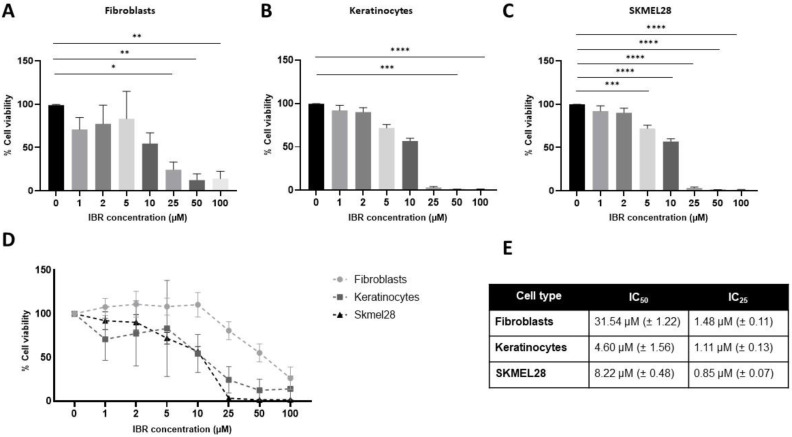
Cytotoxicity of IBR towards human primary skin cells. Human primary skin fibroblasts (**A**), keratinocytes (**B**), and SKMEL-28 cells (**C**) were treated with increasing doses of IBR for 48 h and had their viability assessed using the MTT assay. (**D**) Dose-response cytotoxicity of IBR to the different cell types tested. The data obtained from the MTT assay were used to determine the IC_50_ and IC_25_ of IBR for each cell type (**E**). The data are presented as the mean ± SEM. The data were analyzed using the ANOVA followed by Tukey’s post hoc test for multiple comparisons. * *p* < 0.05; ** *p* < 0.01; *** *p* < 0.001; **** *p* < 0.0001.

The MTT assay was used to evaluate the sensitivity of cells to IBR (Figure 4A–E). Fibroblasts presented lower sensitivity to the drug compared to the other cell types, while keratinocytes were more sensitive to IBR compared to both fibroblasts and the melanoma cell line SKMEL-28. The drug significantly reduced fibroblast viability in concentrations of >50 µM (0 × 50 µM, *p* = 0.0009; 0 × 100 µM *p* < 0.0001) (Figure 4A). The primary epidermal cells presented a significant decrease in viability when treated with >25 µM IBR (*p* = 0.02) (Figure 4B). Cells from the melanoma cell line SKMEL-28 showed a reduction in cell viability starting from 5 µM IBR (*p* = 0.0001) compared to the control (Figure 4C). The viability results were used to calculate the IC_50_ and IC_25_ of IBR in each cell type, the IBR IC_50_ in fibroblasts being 31.54 ± 1.22 µM versus 4.60 ± 1.56 µM in keratinocytes and 8.22 ± 0.48 µM in SKMEL-28 cells. The IC_25_ of IBR was calculated as 1.48 ± 0.11 µM in fibroblasts, 1.11 ± 0.13 µM in keratinocytes, and 0.85 ± 0.07 µM in SKMEL-28 cells (Figure 4D–E).

**Figure 5:**
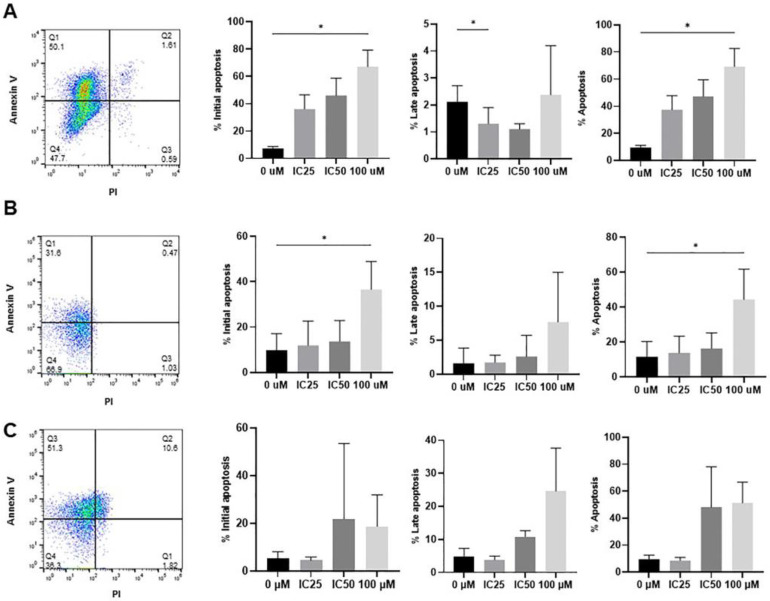
Apoptotic induction by IBR in human primary skin cells. Human primary skin fibroblasts (**A**), keratinocytes (**B**), and SKMEL-28 cells (**C**) were treated with the IC_25_, IC_50_, as well as high (100 µM) IBR doses for 48 h and had their viability assessed using annexin V and PI staining. Representative dot plots are shown on the left. The data are presented as the mean ± SEM. The data were analyzed using the ANOVA followed by Tukey’s post hoc test for multiple comparisons. * *p* < 0.05.

Previous results from our group indicated that IBR induces melanoma cell death through apoptosis induction [20]. Therefore, we also evaluated whether the treatment of cells with IC_50_ and IC_25_ IBR induced annexin V/PI staining. Our results showed that in fibroblasts, there was a trend towards an increase in annexin V+ cells following the treatment with the IC_25_ and IC_50_ IBR doses and a significant increase in the total apoptotic cells upon treatment with 100 µM IBR (Figure 5). Such observations are compatible with apoptotic induction. In keratinocytes, apoptosis was not significantly induced following treatment with the IC_25_ and IC_50_ IBR doses. Considering that IBR inhibits EGFR signaling and that EGFR inhibition promotes keratinocyte cornification, it is possible that the MTT reduction observed resulted from the inhibition of keratinocyte proliferation rather than apoptosis induction. In SKMEL-28 cells, the average total apoptosis increased >4-fold following IC_50_ and 100 µM IBR treatments.

### 3.3. IBR Toxicity to hOSEC Is Dependent on the Administration Route

**Figure 6:**
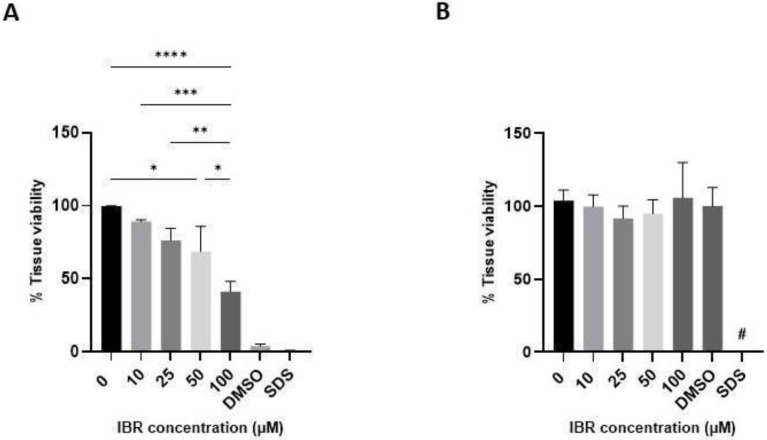
Irritation potential of IBR in hOSEC models. Human organotypic skin explant cultures were treated with increasing doses of IBR. The drug was either added in the tissue culture medium (**A**) or topically administered (**B**). After 48 h, tissue metabolic viability was assessed using the TTC assay. The data are presented as the mean ± SEM. The data were analyzed using the ANOVA followed by Tukey’s post hoc test for multiple comparisons. * *p* < 0.05; ** *p* < 0.01; *** *p* < 0.001; **** *p* < 0.0001; and # *p* < 0.0001 compared to all other groups.

The hOSEC viability test was conducted using the TTC method, which proved superior to the MTT, neutral red (NR), and lactate dehydrogenase (LDH) activity assays for this purpose [21]. When IBR was added in the tissue culture medium for 48 h, the hOSEC models presented a significant loss of tissue viability at concentrations of 50 µM (*p* < 0.05) and 100 µM (*p* < 0.001) compared to untreated controls (Figure 6A). Both DMSO and SDS were used as positive toxicity/irritation controls, and both treatments resulted in a significant reduction in viability compared to the control (*p* < 0.0001). Interestingly, when we repeated both assays by applying IBR on top of hOSEC models, mimicking a topical application, the drug toxicity was reduced. In the TTC assay, only SDS promoted significant tissue toxicity, while topically administered IBR was not toxic up to 100 µM (Figure 6B).

**Figure 7:**
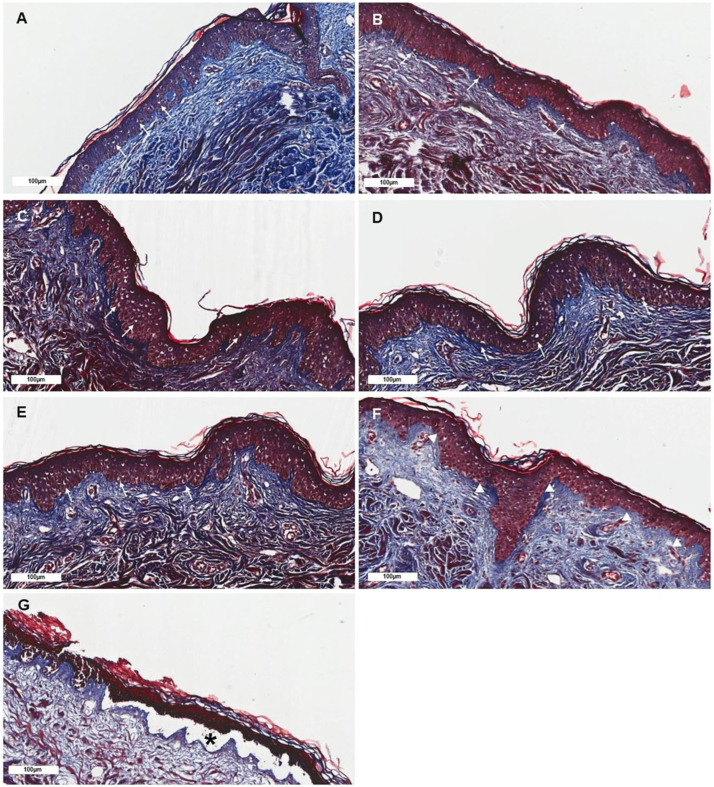
Histological observations of ex vivo skin samples treated with IBR that was added into the tissue culture medium. (**A**) Control group with slight peri-nuclear edema (white arrow) in the epidermis. (**B**) Group treated with 10 µM presenting mild peri-nuclear edema (white arrow) in the epidermis. (**C**) Group treated with 25 µM presenting mild peri-nuclear edema (white arrow) in the epidermis. (**D**) Group treated with 50 µM presenting mild peri-nuclear edema (white arrow) in the epidermis. (**E**) Group treated with 100 µM presenting mild peri-nuclear edema (white arrow) in the epidermis. (**F**) Group treated with DMSO presenting nuclear pyknosis (white arrowhead). (**G**) Group treated with SDS presenting necrotic skin and dermal–epidermal junction separation (asterisk). Scale bar: 100 µm. Masson’s trichrome.

**Figure 8:**
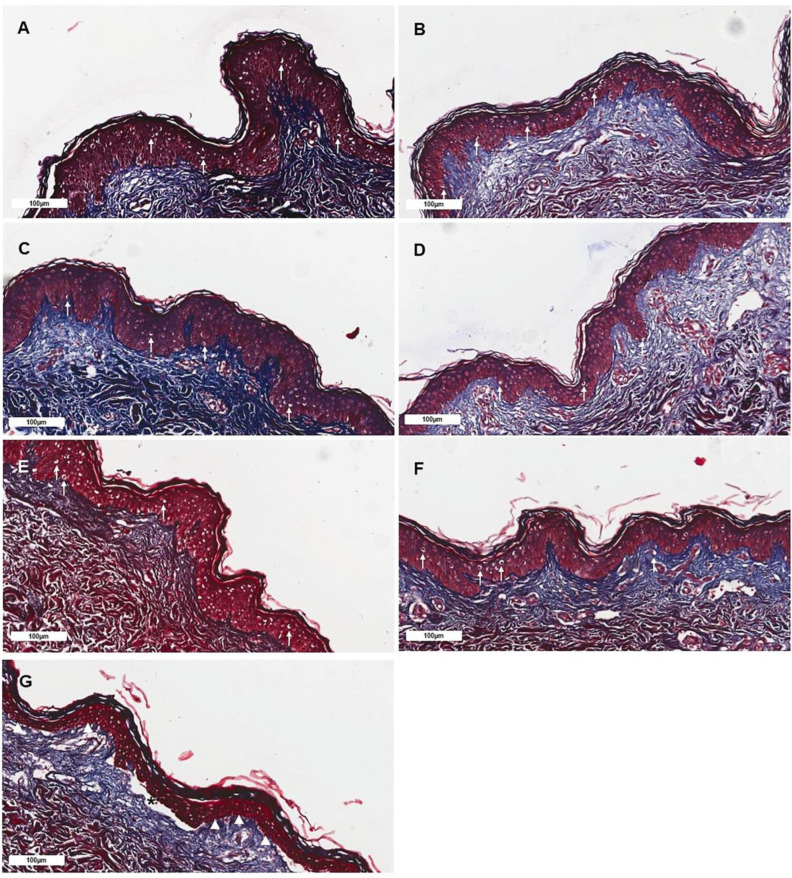
Histological observations of ex vivo skin samples topically treated with IBR. (**A**) Control group with slight peri-nuclear edema (white arrow) in the epidermis. (**B**) Group treated with 10 µM presenting mild peri-nuclear edema (white arrow) in the epidermis. (**C**) Group treated with 25 µM presenting mild peri-nuclear edema (white arrow) in the epidermis. (**D**) Group treated with 50 µM presenting mild peri-nuclear edema (white arrow) in the epidermis. (**E**) Group treated with 100 µM presenting mild peri-nuclear edema (white arrow) in the epidermis. (**F**) Group treated with DMSO presenting nuclear pyknosis (white arrow). (**G**) Group treated with SDS presenting necrotic skin, nuclear pyknosis (white arrowhead), and dermal–epidermal junction separation (asterisk). Scale bar: 100 µm. Masson’s trichrome.

The histological assessment of skin irritation revealed that the hOSEC samples maintained a normal structure in the observation periods of 48 h, with rare structural alterations, such as peri-nuclear edema in the epidermis, cytoplasmic edema, and nuclear pyknosis (Figure 7 and Figure 8). Also, as expected, the SDS-treated samples presented striking alterations regardless of the administration route (tissue culture medium or topical application), with intense intracellular edema and cellular lesions being observed, including necrosis of epidermal cells, peri-nuclear edema (specifically in the epidermis), and nuclear pyknosis. Dermal–epidermal junction alterations were also detected. While DMSO added in the tissue culture medium promoted mild tissue alterations, such as peri-nuclear edema (epidermis), nuclear pyknosis, and necrosis of epidermal cells, when topically applied, the drug presented a lower irritation score. The hOSEC samples exhibited progressive histological alterations with increasing doses of IBR, though they never reached the level of tissue irritation observed in the SDS-treated samples, which displayed significant changes including epidermal cell necrosis, nuclear pyknosis, and separation of the dermal–epidermal junction (Figure 7 and Figure 8, Supplementary Figures S1 and S2; Table 3 and Table 4).

### 3.4. IBR Successfully Permeates the Stratum Corneum

**Figure 9:**
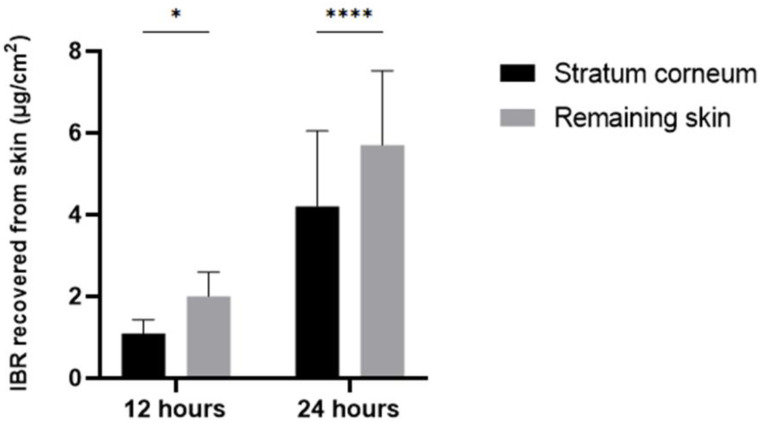
IBR dermatokinetics analysis in human skin. IBR was topically administered in human skin samples. The amount of drug recovered from the stratum corneum and remaining skin after 12 and 24 h was determined using the HPLC method. The data are presented as the mean ± SEM. The data were analyzed using a two-way ANOVA followed by Sidak’s post hoc test for multiple comparisons; * *p* < 0.05; **** *p* < 0.001.

Skin permeation of IBR was analyzed at two different time intervals: 12 and 24 h. Two skin layers were analyzed: the stratum corneum and the remaining skin. For this assay, IBR was formulated in a Carbopol gel, which presented a pH of 5.65 and viscosity of 111.004 Pa.s. This method is considered reliable since it was developed using skin and culture medium as matrices for IBR detection. We observed that IBR was capable of penetrating the stratum corneum after 12 h, being detected both in the stratum corneum and the remaining skin (Figure 9). After 24 h of incubation, the amount of drug that reached both skin layers increased, showing that drug penetration still occurred in this period of observation. The amount of drug observed in the remaining skin was superior to that detected at the stratum corneum after 12 (*p* = 0.0294) and 24 h (*p* < 0.0001). This suggests that IBR has the ability to reach different layers of the skin when topically administered.

## 4. Discussion

IBR was initially developed as a treatment for malignant diseases of B cells, in which BTK is a crucial protein for cell survival and proliferation. Gradually, IBR was found to modulate at least ten other kinases, including enzymes associated with solid tumors [24]. These findings have spurred investigations into the potential of IBR as a treatment strategy for different cancer types, including melanoma [17,20,25].

The analysis of the safety and efficacy of IBR as a treatment option for melanoma is underway both in preclinical and clinical settings. In vitro, it has been consistently shown that IBR induces significant toxicity to leukemic B cells at nanomolar ranges [26]. For skin cancers, IBR has shown promising results as an effective inducer of autophagy in carcinoma [25] and apoptosis in melanoma cell lines at micromolar ranges [20]. Further supporting the clinical potential of IBR for melanoma treatment, our group has recently shown that a high expression of IBR target genes is associated with the enrichment of apoptosis and necrosis pathways in clinical melanoma samples [20]. Nevertheless, IBR failed to promote clinical benefits when systemically administered in treatment-refractory distant metastatic cutaneous melanoma patients during a phase II trial [27]. In this study, the adverse effects (including skin reactions) affected half of the patient cohort, similar to previously published reports [28]. In the long term, IBR has been documented to increase the risk of skin cancer incidence, including both melanoma and non-melanoma skin cancers [29,30]. The low oral bioavailability of IBR [15] coupled with the high doses required to effectively target tumor cells may contribute to both short- and long-term side effects. In this scenario, finding a safe and effective way to deliver IBR directly to melanoma cells might be an effective strategy to guarantee future clinical success.

In the present study, we hypothesized that the topical administration route might be useful to treat non-metastatic melanoma lesions with IBR. Before proposing such a route of administration, we set out to determine the skin toxicity, irritation, and dermatokinetics profile of IBR using ex vivo skin models.

First, we evaluated whether primary skin cells express IBR target genes, such as *EGFR*, *JAK3*, *BTK*, *ITK*, and *ERBB2* [12]. The expression of these genes was determined in skin fibroblasts and keratinocytes and compared to the mRNA levels found in the melanoma cell line SKMEL-28. A variable expression of the tested genes was observed, yet in the same range as the expression levels found in the melanoma cell line.

We conducted cell viability assays on fibroblasts, keratinocytes, and SKMEL-28 cells exposed to different IBR concentrations and observed a positive dose-response relationship in the reduction of cell viability in treated cells, which is in agreement with findings previously documented in the literature [20,25]. In all cell types, IBR seems to reduce cell viability through apoptotic induction, similar to the observations previously made by our group and others [24,29]. Compared to the melanoma cell line SKMEL-28, keratinocytes presented a similar level of sensitivity to the drug, while fibroblasts demonstrated higher resistance to IBR treatment.

Considering that the toxicity of topically administered drugs is heavily influenced by the capacity of penetrating the stratum corneum, we set out to investigate the toxicity of IBR in cultures of organotypic human skin explants, known as hOSECs. We determined IBR’s toxicity and irritation potential towards the skin following two routes of administration: incorporating the drug into the tissue culture medium to mimic systemic IBR delivery and applying the drug topically. Two methods were used: the TTC assay and histological analysis.

The results revealed that topical IBR application did not induce toxicity up to a concentration of 100 µM, whereas IBR addition into the culture medium significantly compromised tissue metabolic activity starting at 50 µM. To evaluate the irritation potential of IBR, we assessed the hOSEC structure using histological analysis to identify alterations induced by IBR treatment. Our results showed that IBR’s toxicity is mostly reduced upon topical administration. At 100 µM, IBR only promoted discrete cellular alterations, comprising peri-nuclear edema (epidermis) and nuclear pyknosis. In contrast, samples treated with the drug added directly into the cell culture medium presented moderate cellular changes that included peri-nuclear edema (epidermis) and nuclear pyknosis at lower IBR concentrations, such as 50 µM.

Complementing our analysis, we have demonstrated that IBR permeates the stratum corneum and reaches the viable epidermis and dermis layers [23]. The evaluated gel demonstrated a suitable pH and viscosity characteristics for topical application, enhancing the spreadability of the formulation on the skin and facilitating the retention of IBR on the skin’s surface [31]. Considering that drug toxicity might be influenced by its dermatokinetics, we analyzed the IBR permeation profile at different time points. Our analysis showed that IBR continuously permeated the stratum corneum up to 24 h, which was the longest time point analyzed. Drug penetration into the remaining skin reached approximately 6 µg/cm^2^ (~13.6 µM IBR), which is in the same range as the IC_50_ concentration of the drug to the SKMEL-28 cell line.

Prior studies from our group have already unraveled, at least in part, the cellular toxicity events promoted by IBR towards melanoma cells [20]. Nevertheless, at this point, little is known about how sensitive to IBR human primary skin cells are. In this study, our findings combined metabolic, molecular, and morphological analyses to shed light into the cellular toxicity processes associated with IBR treatment of normal skin cells. We showed that IBR exerts a dose-dependent toxicity towards keratinocytes and fibroblasts, which involves apoptosis induction. According to our histological analysis, it might be possible that necrosis also occurs, especially at high doses of IBR, given that necrosis-associated cell alterations were observed, such as peri-nuclear edema. Keratinocytes showed to be more sensitive to IBR according to all our assays, which showed a more intense reduction of cellular viability in keratinocytes compared to fibroblasts, as well as more frequent structural alterations in the epidermis compared to the dermis of hOSEC models, regardless of the route of IBR administration. Importantly, even at the highest concentration tested, IBR presented an “Average” irritation score, which is consistent with non-irritant classification in patch tests performed on subjects [22].

hOSEC models represent an ex vivo approach that resembles human skin as closely as possible under in vitro conditions [32]. As recently mentioned by Bouwstra et al. [33], ex vivo human skin is the golden standard for ex vivo percutaneous absorption studies. The adoption of explant models has been very attractive for studies in the dermatological field due to their easy obtention and their undisputable potential to mimic tissue and cellular responses, in addition to reducing dependence on animal testing [34]. hOSEC models can reproduce in vivo conditions, temporarily maintaining the skin tissue viability through a culture medium that provides energy and nutrients for cells to sustain their metabolism. Its main advantage lies in the preservation of the stratum corneum, as well as the presence of all skin cell types. Furthermore, the maintenance of hOSECs in an air–liquid interface makes it possible to study topical and transdermal products. As limitations, hOSEC presents variability between patients, similar to all primary cultures, and its performance is strictly dependent on optimized cell culture conditions [19,34,35]. While the metabolic assessment evaluated the total sample metabolic condition, the histological analysis provided a meticulous scoring of minute alterations presented by cells belonging to different layers of the skin upon drug treatment.

In melanoma treatment, where topical alternatives are available but very limited, developing additional topical strategies that use lower drug doses could lead to milder, locally restricted adverse effects. This approach has the potential to improve patient outcomes by enhancing therapeutic options while minimizing systemic side effects and maximizing patient compliance, which is an important factor in disease treatment [35].

Future studies that comprehensively characterize the cellular and tissue toxicity processes underlying our findings and validate the efficacy of topically administered IBR to kill melanoma cells present in the skin using in vitro and in vivo models will importantly contribute to pave the way for the topical use of IBR. Furthermore, modern drug delivery technologies that boost drug penetration into the stratum corneum, such as nanostructured lipid carriers [17], might contribute to facilitating the topical use of this drug. Additional delivery strategies, including cell-penetrating peptides [36], should also be investigated to reduce off-target effects of IBR, such as the ones we have documented in the epidermises of hOSEC models.

## 5. Conclusions

Taken together, our results indicate that despite promoting discrete cellular and tissue toxicity at specific concentrations, IBR successfully permeates the human skin. Therefore, we can conclude that the topical administration of IBR is possible and might constitute a safe and feasible strategy for local melanoma treatment that deserves to be further investigated in order to replace systemic administration and reduce or even eliminate many of the associated side effects.

## Figures and Tables

**Table 1:** Primer sequences.

Gene	Forward Primer	Reverse Primer
*GAPDH*	ACATCGCTCAGACACCATG	TGTAGTTGAGGTCAATGAAGGG
*EGFR*	TTCAGGAGCTGTACGTGCATT	CACAAGCGCTGTGTACCCT
*JAK3*	GCCTGGAGTGGCATGAGAA	CCCCGGTAAATCTTGGTGAA
*BTK*	TCTGAAGCGATCCCAACAGAA	TGCACGGTCAAGAGAAACAGG
*ITK*	GATCAACTGCCTCCACATTG	GGGCATCACCTCTTAGCACA
*ERBB2*	CGGGGTTCCTTCCCCTAATG	CGGGGTTCCTTCCCCTAATG

**Table 2:** Final tolerance score of ex vivo histological irritation test.

Tolerance	Histological Comparison Between Controls and IBR-Treated Samples
Excellent	A single histological change
Very good	Small histological changes A single structure with a higher rating compared to control
Good	Histological changes At least 2 structures rated higher (2) compared to control
Average	Marked histological changes A single structure is classified as 3 or multiple structural changes classified as 2
Bad	Severe histological changes A single structure is classified as 4 or multiple structural changes classified as 3 or multiple structures that cannot be observed due to other changes

**Table 3:** Classification of histological observations of ex vivo skin samples treated with IBR, which was added into the tissue culture medium.

Treatment	Irritation Score	Intracellular Edema			Cellular Injuries		Nuclear Lesions			Dermal–Epidermal Injury	Inflammatory Cells
		Spongiosis	Acantholysis	Vesicles	Cytoplasmic Edema	Necrosis of Epidermal Cells	Peri-Nuclear Edema (Epidermis)	Intranuclear Edema	Nuclear Pyknosis	Dermal–Epidermal Junction Separation	Exocytosis
Control	Very good	0	0	0	0–1	0	2	0	0–2	0	0
10 µM	Good	0	0	0	0	0–1	2–3	0	2–3	0–2	0
25 µM	Average	0	0	0	0	0–2	3–4	0	2–3	0–2	0
50 µM	Average	0	0	0	0	0–1	3–4	0	2–3	0–2	0
100 µM	Average	0	0	0	0	0	3–4	0	2–3	0	0
SDS	Bad	2–+4	2–+4	0–+4	0–+4	+4–4	0–+4	0–+4	+4–4	+4–4	0–+4
DMSO	Average	0–2	0	0	0–1	0	0	0	2–3	0	0

**Table 4:** Classification of histological observations of ex vivo skin samples treated with topically administered IBR.

Treatment	Irritation Score	Intracellular Edema			Cellular Injuries		Nuclear Lesions			Dermal–Epidermal Injury	Inflammatory Cells
		Spongiosis	Acantholysis	Vesicles	Cytoplasmic Edema	Necrosis of Epidermal Cells	Peri-Nuclear Edema (Epidermis)	Intranuclear Edema	Nuclear Pyknosis	Dermal–Epidermal Junction Separation	Exocytosis
Control	Excellent	0	0	0	0	0	2	0	0	0	0
10 µM	Very good	0–1	0	0	0	0	2–3	0	0–1	0	0
25 µM	Very good	0	0	0	0	0–1	2–3	0	0–1	0	0
50 µM	Very good	0–1	0	0	0	0–1	2	0	1	0	0
100 µM	Average	0	0	0	0	0–1	1–3	0	0–1	0	0
SDS	Bad	0–1	2	2	0	3–4	0–2	0	3–4	3	0
DMSO	Good	0	0	0	0	0–1	1–2	0	1	0	0

## Data Availability

Data can be provided by the authors upon reasonable request.

## Supplementary Materials

The following supporting information can be downloaded at: https://www.mdpi.com/article/10.3390/pharmaceutics16111377/s1, Figure S1: Histological observations of ex vivo skin samples treated with IBR added in the tissue culture medium; Figure S2: Histological observations of ex vivo skin samples treated with IBR added in the tissue culture medium.
