# Supplementary material for: Toxicity and Dermatokinetic Analysis of Ibrutinib in Human Skin Models
Source: Pharmaceutics. 2024 Oct 26;16(11):1377. doi: 10.3390/pharmaceutics16111377 (PMC11597583; doi:10.3390/pharmaceutics16111377)
Supplement: Supplementary file 1 [file pharmaceutics-16-01377-s001.zip › pharmaceutics-3222052-supplementary.pdf]

TOXICITY AND DERMATOKINETIC ANALYSIS OF IBRUTINIB IN HUMAN SKIN MODELS

Supplementary Material

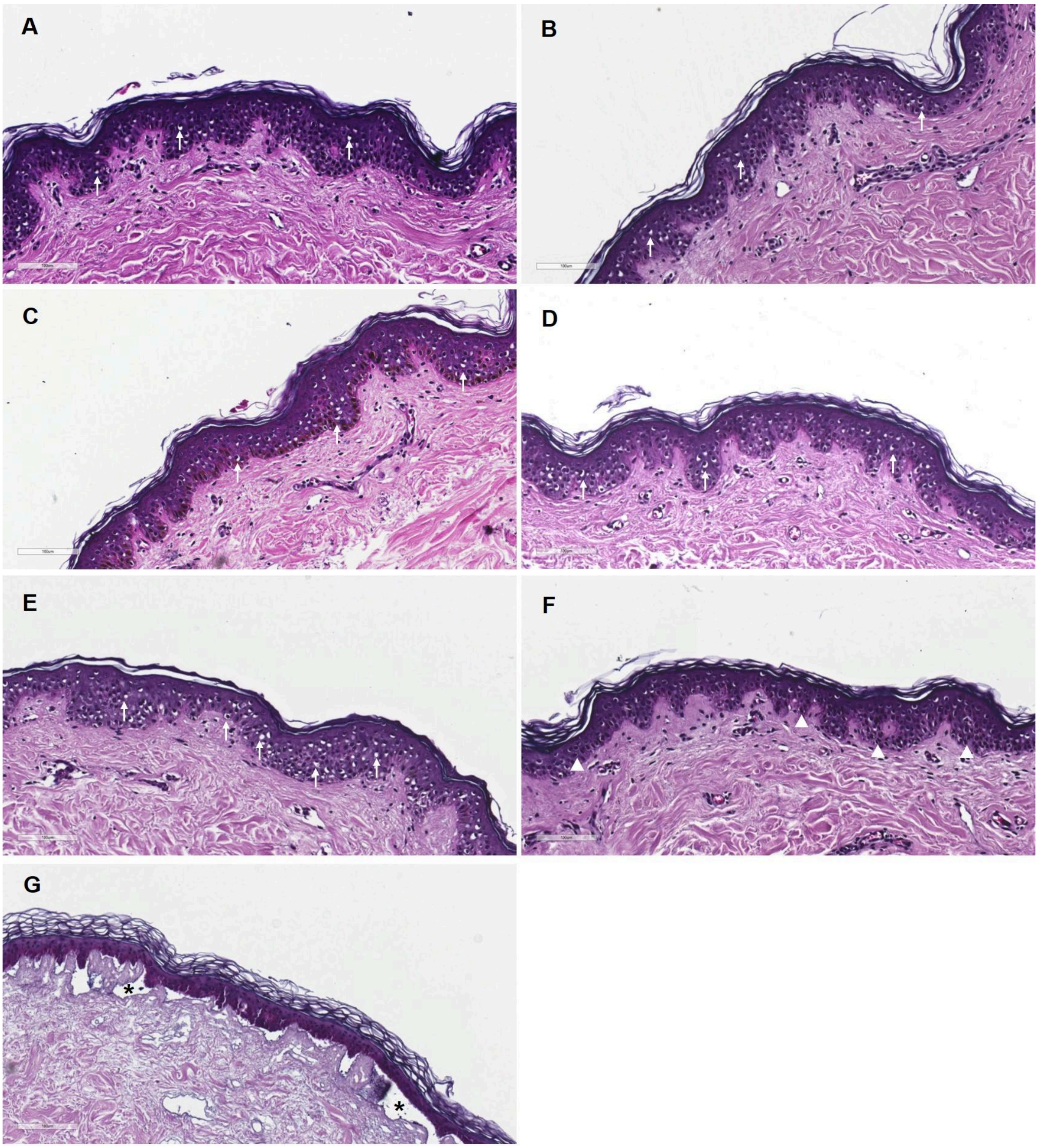

Supplementary Figure 1: Histological observations of ex vivo skin samples treated with IBR added in the tissue culture medium. (A) Control group with slight peri-nuclear oedema (white arrow) in the epidermis. (B) Group treated with 10 µM presenting mild peri-nuclear oedema (white arrow) in the epidermis. (C) Group treated with 25 µM presenting mild peri-nuclear oedema (white arrow) in the epidermis. (D) Group treated with 50 µM presenting mild peri-nuclear oedema (white arrow) in the epidermis. (E) Group treated with 100 µM presenting mild peri-nuclear oedema (white arrow) in the epidermis. (F) Group treated with DMSO presenting nuclear pyknosis (white arrow head). (G) Group treated with SDS presenting necrotic skin and dermal-epidermal junction separation (asterisk). Scale bar: 100 µm. Masson's trichrome.

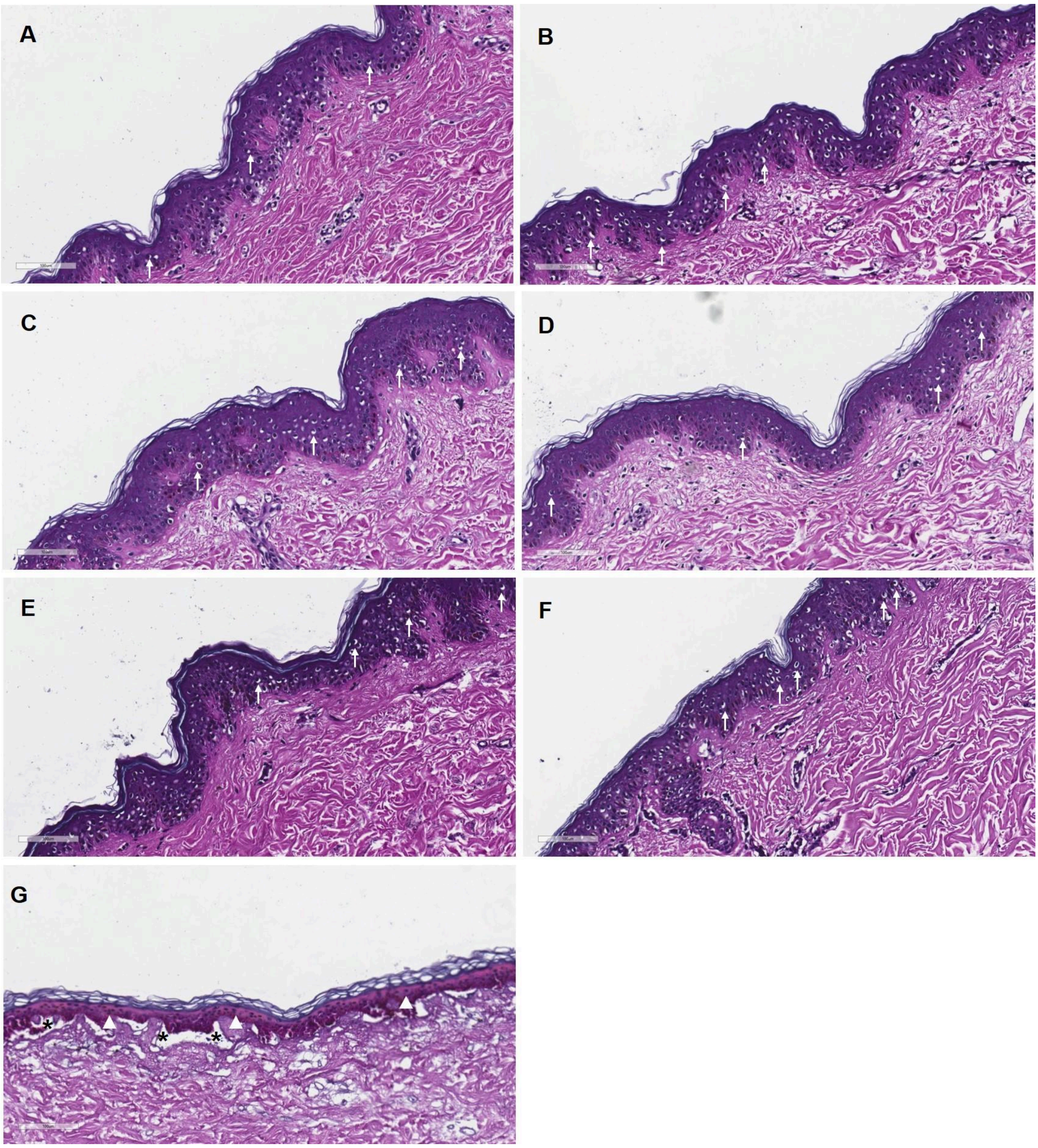

Supplementary Figure 2: Histological observations of ex vivo skin samples treated with IBR added in the tissue culture medium. (A) Control group with few peri-nuclear oedema (white arrow) in the epidermis. (B) Group treated with 10 µM presenting slight peri-nuclear oedema (white arrow) in the epidermis. (C) Group treated with 25 µM presenting few peri-nuclear oedema (white arrow) in the epidermis. (D) Group treated with 50 µM presenting few peri-nuclear oedema (white arrow) in the epidermis. (E) Group treated with 100 µM presenting mild peri-nuclear oedema (white arrow) in the epidermis. (F) Group treated with DMSO presenting few peri-nuclear oedema (white arrow) in the epidermis. (G) Group treated with SDS presenting necrotic skin, nuclear pyknosis (white arrow head), and dermal-epidermal junction separation (asterisk). Scale bar: 100 µm. Masson's trichrome.
